# Different atrophy-hypertrophy transcription pathways in muscles affected by severe and mild spinal muscular atrophy

**DOI:** 10.1186/1741-7015-7-14

**Published:** 2009-04-07

**Authors:** Caterina Millino, Marina Fanin, Andrea Vettori, Paolo Laveder, Maria Luisa Mostacciuolo, Corrado Angelini, Gerolamo Lanfranchi

**Affiliations:** 1 CRIBI Biotechnology Centre, 35121 Padova, University of Padova, Italy; 2 Department of Biology Via Bassi, 35121 Padova, University of Padova, Italy; 3 Department of Neurosciences, Via Giustiniani, 35128 Padova, University of Padova, Italy; 4 Venetian Institute for Molecular Medicine, Via Orus, 35129 Padova, Italy

## Abstract

**Background:**

Spinal muscular atrophy (SMA) is a neurodegenerative disorder associated with mutations of the *survival motor neuron *gene *SMN *and is characterized by muscle weakness and atrophy caused by degeneration of spinal motor neurons. SMN has a role in neurons but its deficiency may have a direct effect on muscle tissue.

**Methods:**

We applied microarray and quantitative real-time PCR to study at transcriptional level the effects of a defective *SMN *gene in skeletal muscles affected by the two forms of SMA: the most severe type I and the mild type III.

**Results:**

The two forms of SMA generated distinct expression signatures: the SMA III muscle transcriptome is close to that found under normal conditions, whereas in SMA I there is strong alteration of gene expression. Genes implicated in signal transduction were up-regulated in SMA III whereas those of energy metabolism and muscle contraction were consistently down-regulated in SMA I. The expression pattern of gene networks involved in atrophy signaling was completed by qRT-PCR, showing that specific pathways are involved, namely IGF/PI3K/Akt, TNF-α/p38 MAPK and Ras/ERK pathways.

**Conclusion:**

Our study suggests a different picture of atrophy pathways in each of the two forms of SMA. In particular, p38 may be the regulator of protein synthesis in SMA I. The SMA III profile appears as the result of the concurrent presence of atrophic and hypertrophic fibers. This more favorable condition might be due to the over-expression of MTOR that, given its role in the activation of protein synthesis, could lead to compensatory hypertrophy in SMA III muscle fibers.

## Background

Spinal muscular atrophy (SMA) is a neurodegenerative disorder with progressive paralysis caused by the loss of motor neurons. Mutations of both alleles of the telomeric *survival motor neuron *(*SMN*) gene *SMN1 *are correlated with the development of SMA [1]. The SMA phenotype can be influenced by the variable copy number of the paralogous centromeric gene *SMN2 *[2-4] which, lacking exon 7, codifies a protein with reduced self-oligomerization and stability [5,6].

SMN is a ubiquitously expressed protein complex implicated in a variety of processes, including the formation and function of neuromuscular junctions [7,8]. Deficiency of the SMN protein may have a specific effect within the motor neuron, connected to RNA metabolism or transcription, which impairs the biogenesis of axons. SMN may be important in the muscle cell itself, and its lack might lead to faulty signaling from skeletal muscle [9-11]. The effect of *SMN *gene mutations in the degeneration of muscle fibers, independent of motor neurons, is supported by results obtained in mice with a deletion of *SMN *exon 7 restricted to skeletal muscle [10]. In *Drosophila melanogaster *a sarcomeric SMN protein has been demonstrated, implicating a muscle-specific function and underlining the importance of this tissue in modulating the severity of SMA phenotype [11].

The main pathological trait of SMA muscles is atrophy, albeit with a variable severity. Many studies have identified elements of the signaling cascades leading to muscle atrophy [12-16]. We compared the expression signatures of human muscles affected by the two extreme forms of SMA (I and III) to understand which genes, other than *SMN*, are involved in muscle-specific SMA pathways and to understand the mechanisms leading to and sustaining atrophy in different forms of SMA.

## Methods

### Characterization of patients with SMA and SMA samples

For this study we analyzed muscle biopsies and genomic DNA from peripheral blood of four patients with SMA I and five patients with SMA III from the Neuromuscular Bank of the University of Padova. The Bank has been approved by the Ethical Committee of the University of Padova in compliance with the Helsinki Declaration. The clinical traits of the patients are summarized in Additional file 1. Atrophy and hypertrophy values of muscle biopsies were obtained by comparing the diameters of random selections of SMA muscle fibers with normal muscle of similar age (see Additional file 2, Extended Methods for details of the methodology).

### Genomic analysis of patients with SMA

Genomic DNA was isolated from whole blood by the salting-out procedure [17]. To identify the presence of *SMN1 *gene deletions in exon 7 and 8, PCR amplifications and restriction enzyme digestions were carried out according to the method proposed by Van der Steege et al [18]. Deletions of exon 5 in the *NAIP *gene were also checked by performing multiplex PCR as proposed by Roy et al [19]. To identify the number of the *SMN2 *gene copies we employed a fluorescence-based competitive PCR assay previously described by Scheffer et al [20], with modifications. Primer sequences and experimental conditions used in multiplexed PCR tests are reported in Additional file 2.

### Microarray experiments

We used a microarray platform containing 4670 muscle-specific 3'-cDNA fragments that is deposited and described in the GEO database (GPL2011) [21]. Patient and control biopsies were taken from quadriceps femoralis muscles. Total RNA was purified by the TRIZOL protocol and quality checked by Agilent Bioanalyzer 2100. One microgram of each RNA sample was linearly amplified using the Amino Allyl MessageAmp II aRNA Amplification Kit (Ambion) and labeled with fluorescent amino allyl-dUTP (Cy3 or Cy5, GE Healthcare). Patient and control cDNAs, labeled with different dyes, were competitively hybridized to microarray platforms. Each microarray experiment was done in duplicate, inverting the labeling dye. Microarrays were read in a Perkin-Elmer LITE confocal laser scanner, and images were analyzed with QuantArray Software (GSI Lumonics, Ottawa, Canada). Statistical analysis of data was performed as detailed in Additional file 2. Expression datasets are compiled according to the standards proposed by the Microarray Gene Expression Data Society and are available at the GEO database with ID series GSE8359.

### Real-time quantitative PCR

Real-time quantitative PCR (qRT-PCR) was carried out using the SYBRTM Green chemistry with GeneAmp 5700 Sequence Detection System (Applied Biosystems). The sequences of gene-specific primers and experimental conditions are reported in Additional file 2.

## Results

### Genomic characterization of patients with SMA

We demonstrated a diagnostic deletion of exon 7 and 8 of the *SMN1 *gene in all patients with SMA I and III. For the *SMN2 *gene we found two copies in all patients with SMA I and in a single patient with SMA III, and three or four copies in the remaining four patients with SMA III. We also studied the *NAIP *gene, since deletions in this gene have been associated with SMA [19]. No variation in the *NAIP *gene structure was found in any of the SMA genomes. Table 1 summarizes the results of our genomic studies.

**Table 1:** Genotype of SMA patients.

**Biopsy code**	**SMA** **type**	**Sex**	**Age at biopsy** **(years)**	***SMN1*** **Deletion**	***SMN2*** **copy number**	***NAIP*** **deletion**
**SMA I samples**						

A	1A	M	0,2	7,8 ex.	2	NO

B	1B	F	0,3	7 ex.	2	NO

C	1B	M	0,5	7,8 ex.	2	NO

D	1B	F	0,9	7,8 ex.	2	NO

**SMA I control**						

Cont. 1	NO	M	0,6	NO	-	NO

**SMA III samples**						

E	3A	M	7	7 ex.	3	NO

F	3A	F	7	7,8 ex.	2	NO

G	3A	F	8	7,8 ex.	3	NO

H	3A	F	7	7,8 ex.	4	NO

I	3A	F	11	7,8 ex.	4	NO

**SMA III control**						

Cont. 2	NO	M	8	NO	-	NO

High-molecular weight genomic DNA was obtained from all patients with SMA type I and III enrolled for this study. This was used to control for the presence of *SMN1 *and *NAIP *gene deletions and *SMN2 *copy number. Genomic DNA from age-matched unaffected donors was used to perform parallel control analyses.

### Expression profiling

**Figure 1:**
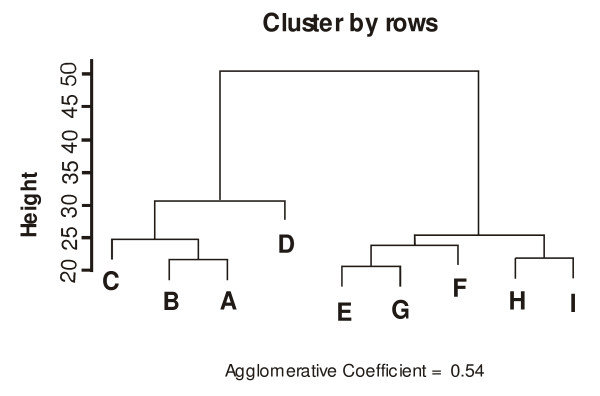
**Hierarchical cluster analysis of SMAI and III profiles**. This dendrogram was constructed with the expression profiles of SMA muscle biopsies using the complete linkage cluster method and the Euclidean distance measure. Patients with SMA are clearly divided into two groups corresponding to the clinical classification: cluster 1 for the more severe type I and cluster 2 for the milder type III.

RNA from each SMA muscle sample was competitively hybridized with age-matched muscle control samples to microarrays. The differential gene expression profiles were subjected to hierarchical clustering, resulting in the patients with SMA being classified in two different groups corresponding to the clinical categorization (Figure 1). This suggests a different gene profile for each muscle atrophic condition (SMA I and III). We determined the concordance between patient classification resulting from microarrays and clinical data with prediction analysis for microarrays (PAM) [22]. The cross-validation test showed that each patient is assigned to the correct clinical class by expression signature. In particular, the transcription level of only 22 significant genes appears to be sufficient to discriminate between SMA I and III profiles, with a misclassification error of 0 (Additional file 3).

The expression data were then analyzed with significance analysis of microarrays for the identification of differentially expressed genes. We only analyzed transcripts with a false discovery rate equal to 0, and with values that showed ratios >0.7 or <-0.7. To study in detail the specific alterations in gene expression caused by the atrophic condition in different SMA muscles we analyzed four different groups of expression data: a) 33 genes that are differentially expressed in patients with SMA I in comparison with controls (10 up- and 23 down-regulated, Additional file 4); b) 10 genes differentially expressed in SMA III in comparison with controls (seven up- and three down-regulated, Additional file 5); c) 46 genes differentially expressed between SMA I and SMA III (15 up- and 31 down-regulated, Additional file 6); d) 19 genes differentially expressed in both SMA I and SMA III in comparison with normal controls (12 up- and seven down-regulated, Additional file 7). These genes have been classified with FatiGO into a number of functional categories: cellular metabolism, muscle contraction, signal transduction, transport, RNA metabolism, molecular recognition system, immune/defense response, cell cycle and others. The similarities in the functional classification of the four groups of differentially expressed transcripts suggest that there should be few metabolic mechanisms involved in the pathological status of SMA muscles.

The analysis of differentially expressed genes in patients with SMA III (Additional file 5) shows a reduced number of altered genes, mainly involved in signal transduction. This suggests proximity of SMA III to the normal muscle condition. Also, the comparison of expression profiles of SMA I and III (Additional file 6) demonstrates that the differences are entirely due to altered genes in SMA I muscles. It is interesting to note that the lists of differentially expressed genes shown in Additional files 4 and 6 are similar, and that there is also good agreement between these lists and the discriminate genes obtained with PAM analysis (Additional file 3).

Finally, Additional file 7 lists deregulated genes that are shared by the two classes of SMA. As can be seen, these are the same genes listed in Additional file 5. Therefore patients with SMA I and III seem to share all those genes whose expression differentiates SMA III muscles from normal controls.

Many transcripts involved in glucose and glycogen metabolism were found to be down-regulated, mainly in SMA I (Additional file 4). mRNAs encoding several enzymes catalyzing different steps of glycolysis, the tricarboxylic cycle and oxidative phosphorylation were reduced. The impairment of cell energy systems seems to be distinctive of the severe disease. Genes implicated in muscle contraction were consistently deregulated, mainly in SMA I samples. Among this class we found genes that codify proteins of the Z disc such as *nebulin *(up-regulated), *desmin *and *titin *(down-regulated) and titin-associated such as *MYBP-C *(down-regulated). At the same time, some genes regulated by *MYBP-C *such as *MYH 1 *and *7 *showed a reduced expression. The analysis also showed a significant under-expression of *CKM*.

qRT-PCR was applied to validate the microarray results. The following transcripts were studied: *Glutathione peroxidase 4 *(*GPX4*) and *Muscle creatine kinase *(*CKM*), that resulted respectively in over and under-expression in SMA I; *Complement factor H *(*CFH*), that resulted in over-expression in both SMA classes. The results confirm those obtained with microarray, and also revealed the under-expression of the *CKM *transcript in SMA III. This is probably due to the higher sensitivity of qRT-PCR technology with respect to microarray [23].

### Molecular pathways of atrophy in SMA I and SMA III muscles

In SMA muscles we monitored the principal pathways involved in atrophic conditions, namely the IGF/PI3K/Akt, TNF-α/p38 MAPK and Ras/ERK pathways. The results are summarized in Table 2 and indicate a different picture for SMA I and III. The analysis of transcriptional profiles showed an under-expression of the *eukaryotic translation initiation factor 4E binding protein 3 *gene (*eIF4EBP3*) only in SMA I, while in other studies the homologous gene *eIF4EBP *was indicated as over-expressed [24]. To study this discrepancy, we decided to probe by qRT-PCR the mRNA levels of some of the principal members of IGF/PI3K/Akt pathway, namely *IGF-1R, FRAP1 (MTOR), FOXO, eIF4EBP1 *and *FBOX32 (Atrogin-1)*. The patients with SMA I showed over-expression of *IGF-1R *and *FOXO*, and under-expression of *FBOX32 *and *eIF4EBP1*, confirming the microarray data. Conversely, in SMA III samples there is a general under-expression of this group of transcripts, with the exception of *MTOR*. Therefore, *MTOR *over-expression might result in the more favorable SMA III phenotype due to its positive signal for protein synthesis activation.

**Table 2:** Real-time quantitative PCR analysis.

	**SMA I**	**SMA III**
**Gene**	**Expression value**	**Expression value**
*IGF-1R*	+3.2	-2.9

*FRAP1* *(MTOR)*	1	+2.8

*FOXO3A*	+1.3	-1.6

*eIF4EBP1*	-1.4	-1.7

*FBOX32* *(Atrogin-1)*	-1.6	-1.7

*TRAF2*	1	+4.2

*MAPK14* *(p38)*	-1.25	+1.6

*RRAS2*	-2	-2.5

*MAPK1* *(ERK)*	+1.2	+2.4

Expression ratios of selected intracellular signaling molecules of atrophy and hypertrophy pathways tested in affected SMAI and III muscles versus control muscles (*p *< 0.05).

Many studies have demonstrated the important role played by *tumor necrosis factor alpha *(*TNF-α*) in regulating muscle size through apoptosis [25]. The *MAPK14 (p38*) gene belongs to TNF-α/p38 MAPK pathway and regulates MAP kinase interacting serine/threonine kinase (MNK1/2). *MNK2 *is down-regulated in patients with SMA I, like *IL-32*, a positive regulator of p38 and TNF-α. Finally *TXN*, a negative regulator of MEKK5 (ASK1) that is upstream of p38, is even more up-regulated in SMA I samples. Considering all these data and the notion that MNK1/2 phosphorylates eIF4E [26], we monitored the TNF-α/p38 MAPK pathway looking for *TRAF2 *and *MAPK14 (p38)*. SMA I muscles showed an under-expression of *p38 *that may be the protein synthesis regulator. In SMA III there was instead a general up-regulation of these genes.

Finally, we investigated the Ras/Raf/MEK/ERK pathway, testing the expression of two members of this pathway (*RRAS2 *and *ERK*) because ERK, like p38, regulates the MNK1/2 activity. This analysis showed that muscles from both groups of SMA resulted in the same expression profile for these factors: *RAS *is down-regulated and *ERK *is up-regulated.

### Correlation of altered molecular pathways with SMA muscle histopathology

**Figure 2:**
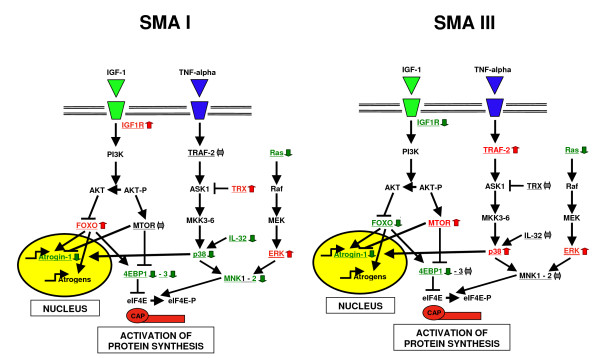
**Summary of expression profiles of SMA I and III muscles of different molecular pathways involved in muscle atrophy/hypertrophy**.

**Figure 3:**
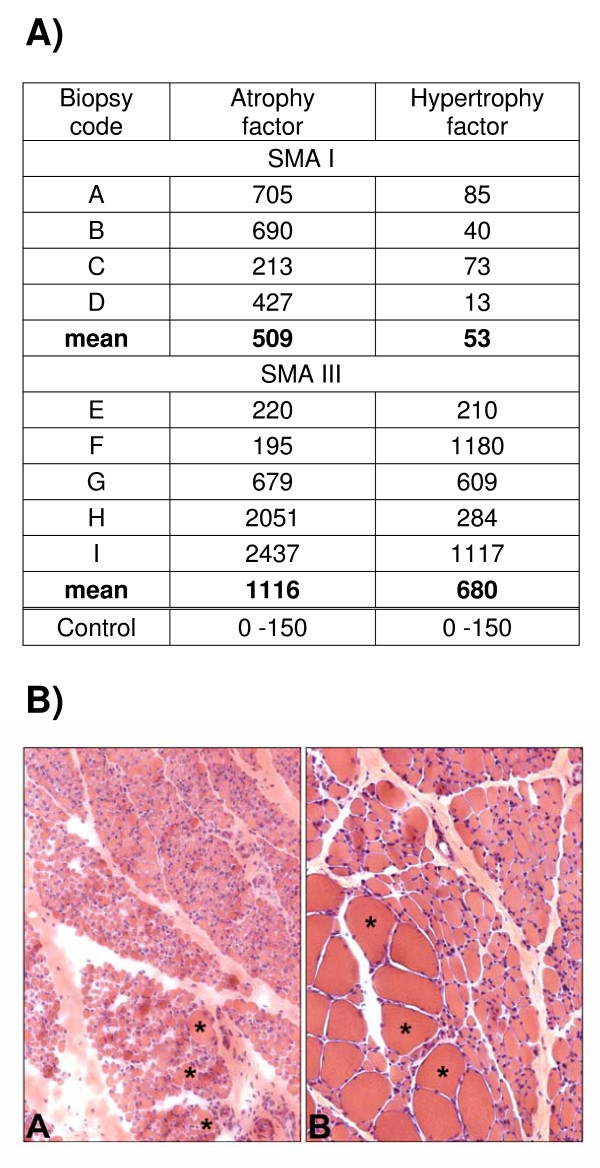
**Panel 1. Fiber size measurements in SMA I and III muscle sections, compared with normal age-matched muscle specimens**. Portions of patient biopsies used for microarray experiments were sectioned and stained with haematoxylin and eosin. Diameters of sample fibers were obtained and atrophy/hypertrophy values were calculated by the methods described in the Additional file 2. Panel 2. Cross-sections of biopsies of quadriceps muscle of patients with SMA I (A) and SMA III (B) photographed at the same enlargement. Muscle from a patient with SMA I shows generalized fiber atrophy and few scattered hypertrophic fibres (asterisks; microscope magnification ×200). Muscle from a patient with SMA III shows groups of atrophic and normotrophic fibers (on the upper-right) and groups of large hypertrophied fibers (asterisks; microscope magnification ×200).

The different expression data obtained from the SMA I and III biopsies (Figure 2), and above all the over-expression of *MTOR*, led us to investigate for the possible presence of hypertrophic fibers in SMA III muscle specimens. Stained sections of the patient biopsies were analyzed and the diameter of muscle fibers was measured in order to calculate atrophy and hypertrophy rate. The result of this investigation (Figure 3) shows that, as expected, both SMA I and III muscles have an increased rate of atrophy in comparison with normal muscle of the same type. The atrophy factor of SMA I is 509 and of SMA III is 1116, the control varying from 0 to 150. On the other hand, fiber measurements show an increased hypertrophy phenotype only for SMA III muscles: the average factor of hypertrophy is indeed 680 when the control varies from 0 to 150.

## Discussion

We studied the differences in the expression signatures of muscles affected by the mild (III) and severe (I) SMA phenotype. All patients with SMA presented an *SMN1 *deletion, and the *SMN2 *copy number was variable and not correlated with the phenotype severity (see Table 1). Therefore, the phenotype of these patients is probably affected by SMN protein level. Deficiency of the SMN protein could lead to this neuromuscular pathology, having a specific role in RNA metabolism of motor neurons or triggering a primary involvement of skeletal muscle which may contribute to motor defects [10]. We investigated these muscle-specific signals by studying the gene expression signatures of SMA muscles. The microarray data outcome gives a different gene expression profile for each of the two forms of SMA. In SMA III muscles the low number of genes presenting an altered expression level suggests a proximity to the normal condition, while in SMA I muscles there is a more complex picture. The hierarchical clustering separates the gene profiles into two groups that correspond to the clinical classification, identifying a shortlist of 22 discriminant transcripts out of 4670 measured by the microarray.

We have compared our results with the list of 'atrogenes' proposed by Goldberg and colleagues as a set of genes that are found commonly deregulated in various types of muscle atrophy in mouse and rat [24,27]. The goal was to understand if the muscular atrophy of patients with SMA involves the same set of genes or a specific transcriptional program. There is a different picture for the two SMA phenotypes. The great majority of genes deregulated in SMA I belonging to the functional classes of energy metabolism and muscle contraction are found among the 'atrogenes' and show a similar expression alteration. With SMA III muscles, this concurrence is found for the genes included in the signal transduction class. This finding indicates once more that SMA I and III muscles are in different phases with distinct transcriptional patterns.

Our study show a complex down-regulation of sarcomeric genes with a reduced metabolic activity more pronounced in the severe form of SMA. mRNAs encoding several enzymes of glycolysis, the tricarboxylic acid cycle and oxidative phosphorylation are reduced (Additional file 4). These changes in gene expression would be expected to suppress the muscle capacity for utilizing glucose and to generally reduce energy production in this tissue. The impairment of the energetic system seems to be distinctive of the more severe SMA I. Indeed, in SMA III muscles we found only two under-expressed transcripts: *isocitrate dehydrogenase 2 *(*IDH2*) and *adenylosuccinate synthase 1 *(*ADSSL1*), enzymes which function downstream of glycolysis. It might be that in the milder form of SMA only the final phases of glucose metabolism are impaired, whereas in the more severe form the impairment is extended also to glycolysis. Genes implicated in muscle contraction were also found consistently deregulated, including some codifying for structural proteins (*nebulin*, *desmin*, *MYH 1 *and *7*) as well as for regulative proteins (*myosin-binding protein C*), confirming the data obtained by others [28].

Both the microarray and qRT-PCR experiments showed the involvement of some molecular pathways that have been already associated with the establishment and maintenance of atrophy in skeletal muscle, namely the IGF/PI3K/Akt pathway and those that regulate MNK1–2 (TNF-α/p38 MAPK and Ras/ERK). Figure 2 summarizes these molecular pathways as determined in the SMA I and III muscles. Akt-1 activity on FOXO and MTOR leads respectively to inhibition or activation of eukaryotic translation initiation factor 4E binding protein (eIF4EBP), determining in turn the depression or the enhancement respectively of cap-dependent protein synthesis [29].

The analysis of these factors, as well as of *FBOX32 *(*Atrogin-1*), gives interesting results, indicating two different conditions for SMA I and III. In SMA I muscle there is a 'prolonged' atrophic pathway where FOXO is over-expressed and potentially able to sustain atrophy. It should be pointed out, however, that our study is done at the transcriptional level and it is known that the FOXO protein needs not only to be up-regulated but also dephosphorylated to enter the myonuclei and activate atrogenes. In contrast, probably by a survival mechanism, there is under-expression of atrogenes downstream of *FOXO *such as *Atrogin-1 *and *eIF4EBP*. This is in agreement with expression profiles obtained for denervated mouse or rat muscles because the expression of these genes in patients with SMA I correlates closely with the profiles of animal muscle in advanced stage of atrophy [30,31]. The inhibition of cap-dependent protein synthesis is probably due to MNK activity regulated by p38 (down-regulated) rather than by ERK. The microarray data corroborate this hypothesis since they show over-expression of *thioredoxin *that inhibits ASK1, which is an upstream positive regulator of p38 [32], and under-expression of *IL32 *that can also activate p38 [33]. Moreover, it was found that the low amount of active MNK resulted in a selective increase of the cap-independent protein synthesis [34]. Therefore, in SMA muscle the under-expression of *MNK *might favor the switching of protein synthesis from cap-dependent to cap-independent.

The situation of atrophy molecular pathways in SMA III muscle is very different. *IGF-1R*, *FOXO*, *Atrogin-1 *and *eIF4EBP1 *are all down-regulated while *MTOR *is up-regulated. These results suggest a possible activation of protein synthesis through the mRNA binding capacity of eIF4 without the inhibiting influence of eIF4EBP. MTOR activation might therefore prevent a general atrophy degeneration, typical of SMA I patients, inducing a compensative skeletal muscle hypertrophy [35]. Indeed, the analysis of the diameter of SMA III fibers shows the presence of a consistent number of hypertrophic fibers that are not detected in SMA I muscle. The histological analysis of SMA I and III muscle has also evidenced atrophy. It is probable therefore that the transcriptome of SMA III muscle is not very different from normal muscle, because it is actually the sum of two opposite transcriptional profiles. Our microarray and qRT-PCR analyses have been carried out with whole muscle biopsies and therefore we have measured the co-existing atrophy and hypertrophy transcriptional profiles with the mutual cancellation of some specific signals, with the exception of MTOR.

## Conclusion

We have studied the transcriptional signature of skeletal muscle tissue affected by spinal muscular atrophy with the significant analysis of very rare human muscle biopsies of the severe SMA type I. Other authors have used this approach to study the transcriptional changes in mouse or rat muscle undergoing atrophy following different physiopathological conditions, but this is the first study focused on human muscle tissues affected by a genetically determined atrophic condition. Our work indicates that SMA I and III muscles are in different phases: the 'prolonged' atrophic condition typical of the SMA I muscle and the co-existence of atrophy and hypertrophy in SMA III muscle. In SMA I muscle there is an extended atrophic pathway with *FOXO *over-expression, the under-expression of downstream FOXO atrogenes and p38 as a probable protein synthesis regulator. Conversely, SMA III muscle appears to have a complex transcriptome arising from co-existence of atrophic and hypertrophic signals. This more favorable condition might be caused by the action of MTOR, whose over-expression could lead to a compensative hypertrophy.

## Competing interests

These or similar data have not been submitted, in whole or in part, for publication elsewhere. The authors have no competing interests to disclose with regard to the manuscript submitted for review.

## Authors' contributions

CM conducted experiments concerning gene expression studies by microarrays and qRT-PCR tests, analysis of expression data and interpretations of results, and contributed to the writing of manuscript draft. MF performed the histopathological characterization of muscle specimens, and helped in the interpretation of expression data. AV conducted the genomic analysis on patients' DNA and interpreted the results. MLM participated in the design of the genomic study of patients' DNA. PL contributed to the interpretation of expression data. CA organized the recruitment of patients, collection of muscle biopsies and clinical histopathological characterization of patients, and contributed to the draft of the manuscript. GL conceived and coordinated the entire study, contributed in the design of experiments and interpretations of results and wrote the manuscript. All authors have read and approved the final manuscript.

## Pre-publication history

The pre-publication history for this paper can be accessed here:



## Supplementary Material

Additional File 1 **Clinical traits**. Clinical traits of SMA patients. Click here for file

Additional File 2 **Extended methods**. This file contains methods for: i) calculation of atrophy and hypertrophy values in children muscle biopsies, ii) SMN2 copy-number assay, iii) statistical analysis of expression data, iv) qRT-PCR. Click here for file

Additional File 3 **Additional Table S2**. This table contains the list of 22 significant transcripts whose differential expression levels allow for a good class prediction with SMA I and III transcriptional profiles. Data have been obtained by PAM analysis. Click here for file

Additional File 4 **Additional Table S3**. This table contains the list of differentially expressed genes in SMA I muscles in comparison to normal age-matched muscle control. Click here for file

Additional File 5 **Additional Table S4**. This table contains the list of differentially expressed genes in SMA III muscles in comparison to normal age-matched muscle control. Click here for file

Additional File 6 **Additional Table S5**. This table contains the list of differentially expressed genes between SMA I and SMA III muscles. Click here for file

Additional File 7 **Additional Table S6**. This table contains the list of genes with an altered expression values in both SMA I and SMA III muscles, in comparison to normal controls. Click here for file
