# Supplementary material for: Different atrophy-hypertrophy transcription pathways in muscles affected by severe and mild spinal muscular atrophy
Source: BMC Med. 2009 Apr 7;7:14. doi: 10.1186/1741-7015-7-14 (PMC2676312; doi:10.1186/1741-7015-7-14)
Supplement: Additional File 1 — Clinical traits. Clinical traits of SMA patients. [file 1741-7015-7-14-S1.doc]

**Table 1.** Clinical characterization of SMA patients

| **SMA type** | **Pt.** | **Sex** | **Family history** | **Symptoms at onset** | **Age and symptoms at biopsy** |
| --- | --- | --- | --- | --- | --- |
| 1A | A | M | Negative | At birth, cefaloematoma, polypnea, hypotonia, extroverted legs | At 2 months, severe hypotonia, contractures of fingers and hip, and *pes equinus varus* |
| 1A | B | F | 1 twin-sister affected | Feeding difficulty | At 5 months, absent limb movements, possible only lateral movements of limbs, abdominal respiration, contractures of fingers and elbows. Died at 5 months of respiratory failure. |
| 1B | C | M | Negative | Hypotonia | At 5 months, severe hypotonia, abdominal respiration and chest deformity, does not control head movements |
| 1B | D | F | Negative | Generalized hypotonia and hypotrophy | At 9 months, unable to sit, severe weakness |
| 3 | E | M | Negative | Neonatal asphyxia, ambulation achieved only at 3 years | At 7 years, waddling gait on tiptoes, Gowers sign, hypotonia, difficulty climbing stairs, speech impairment |
| 3 | F | F | Negative | Since 3 years tiptoe walking | At 7 years, difficulty raising from floor, Gowers sign, Achilles tendon retraction |
| 3 | G | F | Negative | Since infancy difficulty rising from floor | At age 8 years, waddling gait, Gowers sign, knee abduction |
| 3 | H | F | 1 sister affected | At 1 year walked only for 10 meters when supported | At 7 years, difficulty walking, diffuse hypotonia, muscle and tongue atrophy, minipolymyoclonus, knee abduction |
| 3 | I | F | 1 sister affected | Born with hip displasia, walked alone at 1.5 years | At 11 years, difficulty walking only for 100 meters, hyperlordosis, distal muscle atrophy, minipolymyoclonus, tongue fasciculations |
