# Supplementary material for: Different atrophy-hypertrophy transcription pathways in muscles affected by severe and mild spinal muscular atrophy
Source: BMC Med. 2009 Apr 7;7:14. doi: 10.1186/1741-7015-7-14 (PMC2676312; doi:10.1186/1741-7015-7-14)
Supplement: Additional File 2 — Extended methods. This file contains methods for: i) calculation of atrophy and hypertrophy values in children muscle biopsies, ii) SMN2 copy-number assay, iii) statistical analysis of expression data, iv) qRT-PCR. [file 1741-7015-7-14-S2.doc]

**Extended Methods**

##### Calculation of atrophy and hypertrophy values in children muscle biopsies

This statistical approach is particularly useful in detecting the presence of atrophy and hypertrophy that may not be apparent on routine inspection of a muscle biopsy.

The method in adult muscle is based on the fact that nearly all the fibres in the normal adult muscle are between 40 and 80 µm diameter. Among the abnormally small fibres, few fibres in the range of 30-40 µm would have less significance than the same number of fibres in the range of 10 to 20 µm. This was taken into account by multiplying the number of fibres with a diameter between 30 and 40 µm by 1, the number of fibres with a diameter between 20 and 30 by 2, the number of those from 10 to 20 µm by 3, and the number in the group less than 10 µm by 4. These products were then added together and divided by the total number of fibres to put the result on a proportional basis. The resulting number was then multiplied by 1000 to obtain the atrophy and hypertrophy factors. Atrophy and hypertrophy factors were then compared with the upper limit values (normal values: 0-150) for normal muscles^1^.

To adapt this method to muscle biopsies from children, we used the normal values of mean fibre diameters. According to Brooke & Engel^2^ the standard values for average fibre diameters were approximately 16 µm at 1 year of age, increasing by 2 µm for each year to age 5 years and by 3 µm from age 5 to age 9 years. The value at age 10 year was 40 µm. We calculated the normal ranges of fibre diameters for each age of our patients (i.e. 2 months, 5 months, 9 months, 7 years, 8 years, and 11 years) in muscle biopsies from age-matched controls, since these data were not available in the literature. To calculate the atrophy and hypertrophy factors the method was similar to adult muscle except that the multiplying index was attributed to ranges of µm that varied according to the age of patients (e.g. for age 7 years, the mean diameter was 30 µm, the normal range was 20-40 µm, fibres with diameter ranging from 15-20 µm were multiplied by 1, fibres with diameter ranging from 10-15 µm were multiplied by 2; fibres with diameter ranging from 5-10 µm were multiplied by 3).

##### SMN2 copy-number assay

In order to identify the copy number we employed a fluorescence-based competitive PCR assay previously described by Scheffer (2000)^3^. The copy number of *SMN2* was identified in the genomic DNA of SMA patients comparing the amount of *SMN2* exon 7 PCR product with the amount of a co-amplified PCR product of the exon 13 of *retinoblastoma* (*RB1*) gene that is used as reference. Simultaneously, two internal amplicons are generated from plasmids (SMN-IS and RB1-IS) added to PCR reaction for standardisation and monitoring of the amplification efficiency of the competitive PCR reaction. We used genomic DNA as template and R111^4^ plus SMN-is primers to generate the SMN-IS plasmids, while RBex13F and RBIS13 primers^3^ were used to obtain RB-IS plasmids. The SMN-IS and RB-IS are plasmids with *SMN* and *RB1* inserts equal to the genomic amplicons but having internal deletions of 11 and 19 bp respectively. Both ISs are amplified by the same primers as their genomic amplicons but can be distinguished through their different length. Since the genomic *SMN* amplicon is derived from both *SMN1* and *2* genes, the actual *SMN2* PCR product is identified by digesting the PCR reaction in the diagnostic Dra I site of *SMN2* exon 7. Digestion of PCR products is carried out with 5U of Dra I overnight at 37°C. The digested samples are finally separated in an Applied Biosystems ABI PRISM 3100 DNA Sequencer and analysed with the dedicated software (GeneScan, Applied Biosystems). The *SMN2* copy number is obtained by quantification of the digested PCR products, followed by normalization utilizing the genomic standard (*RB*), the internal standards (RB-IS and SMN-IS), and 4 control samples with known *SMN2* copy numbers.

To quantify the various PCR products, the forward primers for *SMN* and for *RB1* exon are labelled with FAM florescent dye. Amplifications were performed in a 30 µl reaction containing 2.5 mM MgCl2, 100 μM of each of the four dNTP, 1 μM each of RBex13F, RBex13R, R111 and X7-Dra primers^3,5^, 1U AmpliTaq Gold polymerase (Applied Biosystem) and 100 ng of genomic DNA in the presence of 2 μl of mixture containing 15,000 molecules/μl of SMN-IS and RB-IS plasmids. An initial denaturing step at 95°C for 10 min was carried out, followed by 24 cycles of 40 sec at 95°C, 50 sec at 57°C, 45 sec at 72°C, and a final extension step at 72°C for 4 min.

**List of primers used in multiplexed PCR for SMN2 copy number determination.**

**SMN-is** 5’-CCTTCCTTCTTTTTGATTTTGTCTAGGAAAATAAAGGAAGTTAAAAA-3’

**R111** 5'-AGACTATCAACTTAATTTCTGATCA-3'

**X7-Dra** 5’-CCTTCCTTCTTTTTGATTTTGTTT-3’

**RBIS13**  5'-TATACGAACTGGAAAGATGCCTATAGTACCACGAATTACAATG-3'

**RBex13F**  5'-ATTACACAGTATCCTCGACA-3'

**RBex13R** 5'-TATACGAACTGGAAAGATGC-3'

##### Statistical analysis of expression data

Intensity values were processed with the gene expression data analysis tool MIDAS (www.tigr.org/software/tm4/). Normalized values were converted to a logarithmic scale. Genes resulting with Log2 expression values >+0.7 and <−0.7 were considered as up-regulated and down-regulated respectively. These threshold values were calculated with pilot experiments where microarray platforms were hybridized with two equal aliquots of control RNA labelled with Cy3 and Cy5 fluorochromes. The log2 fluorescence intensity ratios of 95% of the spots contained in the microarray platform fell between these threshold values. The complete raw and normalized datasets for each microarray experiment have been deposited in the GEO Database (GSE8359). Hierarchical clustering analysis was performed with the statistic web tool MIDAW^6^ (http://muscle.cribi.unipd.it) with complete linkage method and Euclidean distance. Identification of differentially expressed genes was performed with Significance Analysis of Microarray (SAM, http://www-stat.stanford.edu/~tibs/SAM). Class and gene prediction analysis was performed with the Prediction Analysis of Microarrays (PAM, http://www-stat.stanford.edu/~tibs/PAM).

##### Real-time quantitative PCR

Gene-specific primers were selected with Primer3 software (http://frodo.wi.mit.edu/); sequences of contiguous exons were chosen as template for the primer pair to control for the amplification of contaminant genomic DNA. The expression of the following transcripts was studied: *FHL3, GPX4, CKM, CFH*, *FOXO3A, RRAS2, IGF1R, eIF4EBP1, FBOX32 (Atrogin-1), MAPK1 (ERK), MAPK14 (p38), FRAP1 (MTOR), TRAF2*. To evaluate differences in gene expression we chose the relative quantification method, as described by Pfaffl^7^. Values were normalized to the expression of the *RPL32* mRNA that was set as internal reference, since its abundance did not change under our experimental conditions. Normalized ratios were converted in logarithmic scale, and standard deviation was calculated according to published mathematical methodologies^8^.

**Set of primers used for Real-time quantitative PCR**

***Ribosomal protein L32 (RPL32)***

forward: 5'- CATCTCCTTCTCGGCATCA -3'

reverse: 5'- AACCCTGTTGTCAATGCCTC -3'

***Four and a half LIM domains 3 (FHL3)***

forward: 5'- TGCTCTGCAATGACTGCTACTG -3'

reverse: 5'- CCATGTCTGGCCTCCATATTC -3'

***Glutathione peroxidase 4 (GPX4)***

forward: 5'- CCGCTGTGGAAGTGGATGAAGA-3'

reverse: 5'- CACCACGCAGCCGTTCTTGT -3'

***Muscle creatine kinase (CKM)***

forward: 5'- CTGGCACAATGACAACAAGAG -3'

reverse: 5'- GGTGCTGGTTCCACATGA -3'

***Complement factor H (CFH)***

forward: 5'- AGTACCAATGCCAGAACTTGTATC -3'

reverse: 5'- GCTGTCCACCTTAATGCTATGT -3'

***Forkhead box O3A (FOXO3A)***

forward: 5'- TTGATTCCCTCATCTCCACAC -3'

reverse: 5'- TGGCAAAGGGTTTTCTCTGT -3'

***Related RAS viral (r-ras) oncogene homolog 2 (RRAS2)***

forward: 5'- CCATCCAGTTCATCCAGTCCTATT -3'

reverse: 5'- AGCCTTCGCCAGTCCTCATA -3'

***Insulin-like growth factor 1 receptor (IGF1R)***

forward: 5'- CAAGTCCTTCGCTTCGTCAT -3'

reverse: 5'- AGCCTTCGCCAGTCCTCATA -3'

***Eukaryotic translation initiation factor 4E binding protein 1 (eIF4EBP1)***

forward: 5'- AGCCCTTCCAGTGATGAGC -3'

reverse: 5'- GTCCATCTCAAACTGTGACTCTTC -3'

***F-box protein 32 (FBOX32) or Atrogin-1***

forward: 5'- GCTGAATAGCAAAACCAAAACTC -3'

reverse: 5'- CGCTCTTTAGTACTTCCTTTGTGA -3'

***Mitogen-activated protein kinase 1 (MAPK1) or ERK***

forward: 5'- CAAAGAACTAATTTTTGAAGAGACTGC -3'

reverse: 5'- TCCTCTGAGCCCTTGTCCT -3'

***Mitogen-activated protein kinase 14 (MAPK14) or p38***

forward: 5'- CAGTGGCCGATCCTTATGA -3'

reverse: 5'- CACACATGCACACACACTAACA -3'

***FK506 binding protein 12-rapamycin associated protein 1 (FRAP1) or MTOR***

forward: 5'- AGCTGCATGGGGTTTAGGT -3'

reverse: 5'- CCCGAGGGATCATACAGGT -3'

***TNF receptor-associated factor 2 (TRAF2)***

forward: 5'- ATGGCTGACTTGGAGCAGAA -3'

reverse: 5'- CGTCGCCGTTCAGGTAGATA -3'

**References for Extended Methods**

1. Dubowitz V Muscle biopsy. A Practical Approach*.*London: Bailliere & Tindall; 1985.
2. Brooke MH, Engel WK The histographic analysis of human muscle biopsies with regard to fiber types. Children’s biopsies. Neurology 1969;19: 591-605.
3. Scheffer H, Cobben JM, Mensink RG, Stulp RP, van der Steege G, Buys CH. SMA carrier testing-validation of hemizygous SMN exon 7 deletion test for the identification of proximal spinal muscular atrophy carriers and patients with a single allele deletion. Eur J Hum Genet. 2000; 8(2):79-86.
4. Lefebvre S, Burglen L, Reboullet S, Clermont O, Burlet P, Viollet L, Benichou B, Cruaud C, Millasseau P, Zeviani M, et al. Identification and characterization of a spinal muscular atrophy-determining gene. Cell 1995; 80:155-65
5. Van der Steege G, Grootscholten PM, van der Vlies P, Draaijers TG, Osinga J, Cobben JM, Scheffer H, Buyys CH: PCR-based DNA test to confirm clinical diagnosis of autosomal recessive spinal muscular atrophy. Lancet 1995;345:985–986.
6. Romualdi C, Vitulo N, Del Favero M, Lanfranchi G MIDAW: a web tool for statistical analysis of microarray data. Nucleic Acids Res 2005;33: W644-W649.
7. Pfaffl MW A new mathematical model for relative quantification in real-time RT-PCR. Nucleic Acids Res 2001;29: e45.
8. Marino JH, Cook P, Miller KS Accurate and statistically verified quantification of relative mRNA abundances using SYBR Green I and real time RT-PCR. J Immunol Meth 2003;283: 291-306.
