# Supplementary material for: Different atrophy-hypertrophy transcription pathways in muscles affected by severe and mild spinal muscular atrophy
Source: BMC Med. 2009 Apr 7;7:14. doi: 10.1186/1741-7015-7-14 (PMC2676312; doi:10.1186/1741-7015-7-14)
Supplement: Additional File 3 — Additional Table S2. This table contains the list of 22 significant transcripts whose differential expression levels allow for a good class prediction with SMA I and III transcriptional profiles. Data have been obtained by PAM analysis. [file 1741-7015-7-14-S3.doc]

**Table 2.** List of 22 significant transcripts whose differential expression levels allow for a good class prediction with SMA I and III transcriptional profiles. Data have been obtained by PAM analysis with no threshold.

| **ID**  **Array** | **Gene name** | **PAM**  **1**  **score** | **PAM**  **3**  **score** | **SMA biopsy code** | | | | | | | | |
| --- | --- | --- | --- | --- | --- | --- | --- | --- | --- | --- | --- | --- |
|  |  |  |  | **A** | **B** | **C** | **D** | **E** | **F** | **G** | **H** | **I** |
| 2-026D10 | dehydrogenase/reductase (SDR family) member 7 | -1 | 1 | -1.2 | -1.4 | -1.1 | -0.5 | 0.24 | 0.31 | 0.32 | 0.21 | 0.21 |
| 2-028A02 | solute carrier family 24 (sodium/potassium/calcium exchanger) | -1 | 1 | -0.5 | -0.5 | -0.6 | -0.6 | 0.1 | 0.35 | 0.6 | 0.35 | 0.39 |
| 2-002F10 | ATPase inhibitory factor 1 | -1 | 0 | -0.5 | -0.9 | -0.7 | -0.7 | 0.11 | -0 | 0.16 | -0 | 0.06 |
| 2-037D02 | myeloid/lymphoid or mixed-lineage leukemia 5 (trithorax homolog) | 0.5 | -0 | 0.87 | 0.46 | 0.36 | 0.72 | -0.1 | -0.1 | -0.3 | -0.3 | -0.1 |
| 2-009A02 | platelet-activating factor acetylhydrolase | 0.5 | -0 | 0.44 | 0.7 | 0.71 | 0.71 | -0.1 | 0.08 | -0 | 0.09 | -0.2 |
| 2-020G12 | muscle creatine kinase | -0 | 0 | -0.7 | -1.3 | -1.1 | -0.9 | -0 | -0.2 | -0.4 | -0.3 | -0.2 |
| 2-029F08 | chromsome 3 open reading frame 28 | -0 | 0 | -1.2 | -1 | -0.8 | -0.6 | -0.3 | -0.1 | -0.3 | -0.1 | -0.2 |
| 2-030B08 | interleukin 32 | -0 | 0 | -0.9 | -0.8 | -0.8 | -1 | 0.03 | 0.09 | 0.13 | -0.4 | -0.3 |
| 2-038B06 | leucine rich repeat containing 49 | 0.4 | -0 | 0.26 | 0.48 | 0.08 | 0.45 | -0.3 | -0.2 | -0.4 | -0.4 | -0.3 |
| 2-034C03 | UPF3 regulator of nonsense transcripts homolog A | 0.3 | -0 | 0.59 | 1.05 | 1.01 | 0.74 | 0.44 | -0 | 0 | 0.07 | -0 |
| 2-026B03 | GUP1, glycerol uptake/transporter homolog | -0 | 0 | -0.8 | -1.1 | -0.7 | -0.7 | -0 | 0.06 | -0.1 | -0.4 | -0.1 |
| 2-035A10 | ribosomal protein L3-like | -0 | 0 | -0.8 | -1 | -0.9 | -0.6 | -0.1 | 0.07 | -0.3 | -0.3 | -0.3 |
| 2-033A11 | Unknown | 0.3 | -0 | 0.83 | 0.79 | 0.93 | 0.73 | 0.43 | -0.1 | 0.3 | 0.07 | 0.13 |
| 2-011H06 | actin related protein 2/3 complex subunit 2 | -0 | 0 | -0.4 | -0.7 | -0.4 | -0.5 | 0.12 | 0.08 | 0.05 | 0.18 | -0.2 |
| BL-009E04 | Unknown | 0.3 | -0 | 0.8 | 1.65 | 1.1 | 0.89 | 0.36 | -0.2 | 0.4 | 0.33 | 0.05 |
| 2-030D10 | Unknown | 0.3 | -0 | 0.84 | 1.6 | 1.58 | 2.22 | 0.95 | -1.2 | -0.6 | 0.13 | 0.17 |
| BL-005E03 | cereblon | 0.3 | -0 | 0.34 | 0.56 | 0.27 | 0.27 | -0.1 | -0.1 | -0.1 | -0.3 | -0.2 |
| 2-028E08 | angiomotin | -0 | 0 | -0.4 | -0.6 | -0.8 | -1.2 | 0.44 | 0.72 | -0 | -0.2 | 0.1 |
| 2-036B10 | hexokinase 1 isoform HKI-td, | -0 | 0 | -0.5 | -0.8 | -0.8 | -0.7 | -0.1 | 0.32 | -0 | 0.08 | -0.3 |
| 2-015H01 | mitochondrial ribosomal protein L14 | -0 | 0 | -0.6 | -0.3 | -0.5 | -0.4 | 0.2 | 0.05 | -0 | 0.23 | -0 |
| 2-018E09 | myosin, heavy polypeptide 1, skeletal muscle | -0 | 0 | -0.8 | -1.6 | -0.9 | -1.2 | 0.13 | -0.3 | 0.56 | -0.4 | -0.5 |
| 2-029E07 | Unknown | 0.2 | -0 | 1.12 | 0.32 | 1.95 | 1.55 | 0.16 | 0.02 | 0.11 | -0.1 | 0.27 |
