# Supplementary material for: Different atrophy-hypertrophy transcription pathways in muscles affected by severe and mild spinal muscular atrophy
Source: BMC Med. 2009 Apr 7;7:14. doi: 10.1186/1741-7015-7-14 (PMC2676312; doi:10.1186/1741-7015-7-14)
Supplement: Additional File 4 — Additional Table S3. This table contains the list of differentially expressed genes in SMA I muscles in comparison to normal age-matched muscle control. [file 1741-7015-7-14-S4.doc]

**Table 3.** Genes differentially expressed in SMA I muscles in comparison to normal age-matched muscle control.

| **Microarray**  **ID** | **Ref. Seq.** | **functional category and**  **Gene name** | **Gene**  **Symbol** | **Entrez**  **Gene ID** | **SAM**  **Score** | **SMA biopsy code** | | | |
| --- | --- | --- | --- | --- | --- | --- | --- | --- | --- |
|  |  |  |  |  |  | **A** | **B** | **C** | **D** |
| **0ver expressed genes** | | | | | | **Log_2_ ratio intensities** | | | |
|  |  | ***Cellular metabolism*** |  |  |  |  |  |  |  |
| 2-002A09 | NM_002085 | glutathione peroxidase 4 | GPX4 | 2879 | 8 | **0,7** | **0,8** | **1,0** | **0,8** |
|  |  | ***Muscle contraction*** |  |  |  |  |  |  |  |
| 2-016E02 | NM_002469 | myogenic factor 6 | MYF6 | 4618 | 11 | **1,2** | **1,1** | **1,2** | **1,4** |
|  |  | ***Signal transduction*** |  |  |  |  |  |  |  |
| 2-028H10 | NM_000079 | cholinergic receptor, nicotinic, alpha polypeptide 1 | CHRNA1 | 1134 | 7 | **0,8** | **0,7** | **1** | **0,7** |
| 2-038C09 | NM_138822 | peptidylglycine alpha-amidating monooxygenase | PAM | 5066 | 6 | **1,3** | **1,7** | **1,7** | **0,9** |
|  |  | *RNA metabolism* |  |  |  |  |  |  |  |
| 2-029D05 | NM_006925 | splicing factor, arginine/serine-rich 5 | SFRS5 | 6430 | 6 | **1,2** | **1,5** | **2** | **1,1** |
|  |  | ***Molecular recognition system*** |  |  |  |  |  |  |  |
| 2-036H10 | NM_198494 | zinc finger protein 642 | ZNF642 | 339559 | 5 | **1,0** | **1,8** | **1,0** | **0,9** |
|  |  | ***Others*** |  |  |  |  |  |  |  |
| 2-033A11 |  | Unknown |  |  | 10 | **0,8** | **0,8** | **0,9** | **0,7** |
| 2-024C05 | NM_006169 | nicotinamide N-methyltransferase | NNMT | 4837 | 8 | **1,0** | **1,4** | **1,5** | **1,2** |
| 2-030D10 |  | Unknown |  |  | 5 | **0,8** | **1,6** | **1,6** | **2,2** |
| BL-009E04 |  | Unknown |  |  | 5 | **0,8** | **1,6** | **1,1** | **0,9** |
| **Under expressed genes** | | | | | | **Log_2_ ratio intensities** | | | |
|  |  | ***Cellular metabolism*** |  |  |  |  |  |  |  |
| 2-022D11 | NM_004146 | NADH dehydrogenase (ubiquinone) 1 beta subcomplex, 7 | NDUFB7 | 4713 | -9 | **-0,7** | **-0,8** | **-0,9** | **-1,0** |
| 2-001B05 | NM_001916 | cytochrome c-1 | CYC1 | 1537 | -8 | **-1,6** | **-1,4** | **-0,9** | **-1,0** |
| 2-027E11 | NM_005398 | protein phosphatase 1, regulatory (inhibitor) subunit 3C | PPP1R3C | 5507 | -7 | **-1,0** | **-1,4** | **-0,9** | **-1,0** |
| 2-015C07 | NM_001151 | solute carrier family 25 (mitochondrial carrier;ANT) 4 | SLC25A4 | 291 | -6 | **-1,5** | **-2,4** | **-1,7** | **-1,0** |
| 2-002B09 | NM_002168 | isocitrate dehydrogenase 2 (NADP+) | IDH2 | 3418 | -6 | **-1,1** | **-1,6** | **-1,1** | **-1,0** |
| 2-020B09 | NM_152328  NM_199165 | adenylosuccinate synthase-like 1 | ADSSL1 | 122622 | -5 | **-1,0** | **-1,6** | **-1,6** | **-1,0** |
| 2-035G11 | NM_001976 NM_053013 | enolase 3 | ENO3 | 2027 | -5 | **-0,7** | **-1,6** | **-1,2** | **-1,0** |
| 2-036E10 | NM_00216 | isocitrate dehydrogenase 2 (NADP+), mitochondrial | IDH2 | 3418 | -5 | **-0,7** | **-1,6** | **-1,2** | **-1,0** |
|  |  | ***Muscle contraction*** |  |  |  |  |  |  |  |
| 2-023G04 | NM_000257 | myosin, heavy 7, cardiac muscle, beta | MYH7 | 4625 | -7 | **-1,0** | **-0,9** | **-0,8** | **-1,0** |
| 2-020F04 | NM_001824 | muscle creatine kinase | CKM | 1158 | -7 | **-0,9** | **-1,6** | **-1,3** | **-1,0** |
| 2-021F11 | NM_000257 | myosin, heavy 7, cardiac muscle, beta | MYH7 | 4625 | -6 | **-1,4** | **-1,3** | **-1,4** | **-1,0** |
| 2-023H07 | NM_000257 | myosin, heavy 7, cardiac muscle, beta | MYH7 | 4625 | -6 | **-0,9** | **-1,0** | **-1,4** | **-1,0** |
| 2-020G12 | NM_001824 | muscle creatine kinase | CKM | 1158 | -6 | **-0,7** | **-1,3** | **-1,1** | **-1,0** |
| 2-018E09 | NM_005963 | myosin, heavy 1, skeletal muscle | MYH1 | 4619 | -6 | **-0,8** | **-1,6** | **-0,9** | **-1,0** |
| 2-023G08 | NM_206820 | myosin binding protein C, slow type | MYBPC1 | 4604 | -6 | **-0,9** | **-1,3** | **-1,4** | **-1,0** |
| 2-001A02 | NM_001824 | muscle creatine kinase | CKM | 1158 | -5 | **-0,8** | **-1,5** | **-0,9** | **-1,0** |
| 2-020B02 | NM_001927 | desmin | DES | 1674 | -4 | **-0,7** | **-1,3** | **-0,7** | **-1,0** |
|  |  | ***Signal transduction*** |  |  |  |  |  |  |  |
| 2-037B08 | NM_145687  NM_145686  NM_004834 | mitogen-activated protein kinase kinase kinase kinase 4 | MAP4K4 | 9448 | -8 | **-0,9** | **-1,2** | **-1,1** | **-1,0** |
| 2-016E11 | NM_021980 | optineurin | OPTN | 10133 | -6 | **-0,8** | **-1,2** | **-1,3** | **-1,0** |
|  |  | ***Molecular recognition system*** |  |  |  |  |  |  |  |
| 2-005B04 | NM_017812 | coiled-coil-helix-coiled-coil-helix domain containing 3 | CHCHD3 | 54927 | -7 | **-1,2** | **-1,2** | **-1,0** | **-1,0** |
|  |  | ***Immune/Defense response*** |  |  |  |  |  |  |  |
| 2-030B08 | NM_004221 | interleukin 32 | IL32 | 9235 | -9 | **-0,9** | **-0,8** | **-0,8** | **-1,0** |
|  |  | ***Others*** |  |  |  |  |  |  |  |
| 2-022H08 | NM_213720 | chromosome 22 open reading frame 16 | C22orf16 | 400916 | -6 | **-1,4** | **-1,5** | **-1,2** | **-1,0** |
