# Supplementary material for: Different atrophy-hypertrophy transcription pathways in muscles affected by severe and mild spinal muscular atrophy
Source: BMC Med. 2009 Apr 7;7:14. doi: 10.1186/1741-7015-7-14 (PMC2676312; doi:10.1186/1741-7015-7-14)
Supplement: Additional File 5 — Additional Table S4. This table contains the list of differentially expressed genes in SMA III muscles in comparison to normal age-matched muscle control. [file 1741-7015-7-14-S5.doc]

**Table 4.** Genes differentially expressed in SMA III muscles in comparison to normal age-matched muscle control.

| **Microarray**  **ID** | **Ref. Seq.** | **Gene name and**  **functional category** | **Gene**  **Symbol** | **Entrez**  **Gene ID** | **SAM**  **Score** | **SMA biopsy code** | | | | |
| --- | --- | --- | --- | --- | --- | --- | --- | --- | --- | --- |
|  |  |  |  |  |  | **E** | **F** | **G** | **H** | **I** |
| **0ver expressed genes** | | | | | | **Log2 ratio intensities** | | | | |
|  |  | ***Signal transduction*** |  |  |  |  |  |  |  |  |
| 2-010C12 | [NM_014624](http://genome-www4.stanford.edu/cgi-bin/SMD/source/sourceResult?choice=Gene&option=Name&criteria=NM_014624) | S100 calcium-binding protein A6 | [S100A6](http://genome-www4.stanford.edu/cgi-bin/SMD/source/sourceResult?choice=Gene&option=Name&criteria=S100A6) | [6277](http://www.ncbi.nlm.nih.gov/entrez/query.fcgi?db=gene&cmd=Retrieve&dopt=full_report&list_uids=6277) | 6 | **1,13** | **0,5** | **1,0** | **1,6** | **1,1** |
| 2-038C09 | [NM_138822](http://genome-www4.stanford.edu/cgi-bin/SMD/source/sourceResult?choice=Gene&option=Name&criteria=NM_138822,NM_138821NM_138766NM_000919)  [NM_138766](http://genome-www4.stanford.edu/cgi-bin/SMD/source/sourceResult?choice=Gene&option=Name&criteria=NM_138822,NM_138821NM_138766NM_000919)  [NM_000919](http://genome-www4.stanford.edu/cgi-bin/SMD/source/sourceResult?choice=Gene&option=Name&criteria=NM_138822,NM_138821NM_138766NM_000919) | peptidylglycine alpha-amidating monooxygenase | [PAM](http://genome-www4.stanford.edu/cgi-bin/SMD/source/sourceResult?choice=Gene&option=Name&criteria=PAM) | [5066](http://www.ncbi.nlm.nih.gov/entrez/query.fcgi?db=gene&cmd=Retrieve&dopt=full_report&list_uids=5066) | 5 | **1,14** | **0,2** | **0,6** | **1,0** | **0,6** |
|  |  | ***Transport*** |  |  |  |  |  |  |  |  |
| 2-030H05 | [NM_006407](http://genome-www4.stanford.edu/cgi-bin/SMD/source/sourceResult?choice=Gene&option=Name&criteria=NM_006407) | ADP-ribosylation-like factor 6 interacting protein 5 | ARL6IP5 | [10550](http://www.ncbi.nlm.nih.gov/entrez/query.fcgi?db=gene&cmd=Retrieve&dopt=full_report&list_uids=10550) | 5 | **0,9** | **0,1** | **0,8** | **1,1** | **0,9** |
|  |  | Molecular recognition system |  |  |  |  |  |  |  |  |
| 2-036H10 | [NM_198494](http://genome-www4.stanford.edu/cgi-bin/SMD/source/sourceResult?choice=Gene&option=Name&criteria=NM_198494) | zinc finger protein 642 | [ZNF642](http://genome-www4.stanford.edu/cgi-bin/SMD/source/sourceResult?choice=Gene&option=Name&criteria=ZNF642) | [339559](http://www.ncbi.nlm.nih.gov/entrez/query.fcgi?db=gene&cmd=Retrieve&dopt=full_report&list_uids=339559) | 5 | **1,0** | **1,8** | **1,0** | **0,9** | **0,6** |
|  |  | Immune/Defense response |  |  |  |  |  |  |  |  |
| 2-011B07 | NM_002113 | complement factor H, | CFH | 3075 | 8 | **0,92** | **0,5** | **1,0** | **0,8** | **1,0** |
| 2-029D06 | [NM_201442](http://genome-www4.stanford.edu/cgi-bin/SMD/source/sourceResult?choice=Gene&option=Name&criteria=NM_201442,NM_001734)  [NM_001734](http://genome-www4.stanford.edu/cgi-bin/SMD/source/sourceResult?choice=Gene&option=Name&criteria=NM_201442,NM_001734) | complement component 1, s subcomponent | [C1S](http://genome-www4.stanford.edu/cgi-bin/SMD/source/sourceResult?choice=Gene&option=Name&criteria=C1S) | [716](http://www.ncbi.nlm.nih.gov/entrez/query.fcgi?db=gene&cmd=Retrieve&dopt=full_report&list_uids=716) | 5 | **1,1** | **0,3** | **0,6** | **1,1** | **1,0** |
|  |  | ***Others*** |  |  |  |  |  |  |  |  |
| 2-036A04 |  | Unknown |  |  | 5 | **1,28** | **0,3** | **1,1** | **1,5** | **0,8** |
| **Under expressed genes** | | | | | | **Log2 ratio intensities** | | | | |
|  |  | ***Cellular metabolism*** |  |  |  |  |  |  |  |  |
| 2-002B09 | [NM_002168](http://genome-www4.stanford.edu/cgi-bin/SMD/source/sourceResult?choice=Gene&option=Name&criteria=NM_002168) | isocitrate dehydrogenase 2 (NADP+), | [IDH2](http://genome-www4.stanford.edu/cgi-bin/SMD/source/sourceResult?choice=Gene&option=Name&criteria=IDH2) | [3418](http://www.ncbi.nlm.nih.gov/entrez/query.fcgi?db=gene&cmd=Retrieve&dopt=full_report&list_uids=3418) | -4 | **-0,9** | **0** | **-0,8** | **-1,1** | **0,5** |
|  |  | Signal transduction |  |  |  |  |  |  |  |  |
| 2-016E11 | [NM_021980](http://genome-www4.stanford.edu/cgi-bin/SMD/source/sourceResult?choice=Gene&option=Name&criteria=NM_021980) | optineurin | [OPTN](http://genome-www4.stanford.edu/cgi-bin/SMD/source/sourceResult?choice=Gene&option=Name&criteria=OPTN) | [10133](http://www.ncbi.nlm.nih.gov/entrez/query.fcgi?db=gene&cmd=Retrieve&dopt=full_report&list_uids=10133) | -4 | **-0,6** | **0** | **-0,8** | **-0,7** | **0,9** |
|  |  | ***Others*** |  |  |  |  |  |  |  |  |
| 2-001F01 | [NM_213720](http://genome-www4.stanford.edu/cgi-bin/SMD/source/sourceResult?choice=Gene&option=Name&criteria=NM_213720) | chromosome 22 open reading frame 16 | [C22orf16](http://genome-www4.stanford.edu/cgi-bin/SMD/source/sourceResult?choice=Gene&option=Name&criteria=C22orf16) | [400916](http://www.ncbi.nlm.nih.gov/entrez/query.fcgi?db=gene&cmd=Retrieve&dopt=full_report&list_uids=400916) | -4 | **-0,5** | **0** | **-0,8** | **-0,7** | **0,7** |
