# Supplementary material for: Different atrophy-hypertrophy transcription pathways in muscles affected by severe and mild spinal muscular atrophy
Source: BMC Med. 2009 Apr 7;7:14. doi: 10.1186/1741-7015-7-14 (PMC2676312; doi:10.1186/1741-7015-7-14)
Supplement: Additional File 6 — Additional Table S5. This table contains the list of differentially expressed genes between SMA I and SMA III muscles. [file 1741-7015-7-14-S6.doc]

**Table 5.** Genes differentially expressed between SMA I and SMA III muscles.

| **Microarray**  **ID** | **Ref. Seq.** | **Gene name and**  **functional category** | **Gene**  **Symbol** | **Entrez**  **Gene ID** | **SAM**  **Score** | **SMA I biopsy code** | | | | **SMA III biopsy code** | | | | |
| --- | --- | --- | --- | --- | --- | --- | --- | --- | --- | --- | --- | --- | --- | --- |
|  |  |  |  |  |  | **A** | **B** | **C** | **D** | **E** | **F** | **G** | **H** | **I** |
| **0ver expressed genes** | | | | | | **Log_2_ ratio intensities** | | | | | | | | |
|  |  | ***Cellular metabolism*** |  |  |  |  |  |  |  |  |  |  |  |  |
| 2-009A02 | NM_000430 | platelet-activating factor acetylhydrolase | PAFAH1B1 | [5048](http://www.ncbi.nlm.nih.gov/entrez/query.fcgi?db=gene&cmd=Retrieve&dopt=Graphics&list_uids=5048) | 7 | 0.4 | **0.7** | **0.7** | **0.7** | -0.1 | 0.1 | 0 | 0.1 | -0.2 |
| 2-002A09 | NM_002085 | glutathione peroxidase 4 | GPX4 | [2879](http://www.ncbi.nlm.nih.gov/entrez/query.fcgi?db=gene&cmd=Retrieve&dopt=full_report&list_uids=2879) | 3 | **0.7** | **0.8** | **1,0** | **0.8** | 0.3 | 0.2 | 0.2 | **0.8** | 0.1 |
|  |  | *Muscle contraction* |  |  |  |  |  |  |  |  |  |  |  |  |
| 2-002B11 | NM_006471 | myosin regulatory light chain | MRCL3 | 10627 | 4 | **0.8** | **1.1** | **0.8** | 0.2 | 0.2 | 0 | 0 | -0.1 | -0.1 |
| 2-018C06 | NM_004543 | nebulin | NEB | 4703 | 3 | **0.7** | 0.2 | **1.5** | **1.3** | 0.2 | 0.2 | 0.1 | **-1,0** | **-0.7** |
|  |  | ***Signal transduction*** |  |  |  |  |  |  |  |  |  |  |  |  |
| 2-007H01 | NM_003329 | thioredoxin | TXN | 7295 | 4 | **0.9** | **1.2** | **1,0** | 0.4 | 0.2 | 0.1 | 0.1 | 0.3 | 0.4 |
| 2-028H10 | NM_000079 | cholinergic receptor, nicotinic, alpha polypeptide 1 (muscle) precursor | CHRNA1 | [1134](http://www.ncbi.nlm.nih.gov/entrez/query.fcgi?db=gene&cmd=Retrieve&dopt=full_report&list_uids=1134) | 3 | **0.8** | **0.7** | **1,0** | **0.7** | **0.7** | 0.1 | 0.3 | 0.4 | 0.2 |
|  |  | ***Transport*** |  |  |  |  |  |  |  |  |  |  |  |  |
| 2-024G05 | NM_002959 | sortilin 1 preproprotein | SORT1 | 6272 | 3 | 0.6 | **0.8** | **0.8** | **0.8** | 0.5 | -0.2 | 0.5 | 0.3 | 0.1 |
|  |  | ***RNA metabolism*** |  |  |  |  |  |  |  |  |  |  |  |  |
| 2-034C03 | NM_080687 NM_023011 | regulator of nonsense transcripts homolog A | UPF3A | [65110](http://www.ncbi.nlm.nih.gov/entrez/query.fcgi?db=gene&cmd=Retrieve&dopt=Graphics&list_uids=65110) | 5 | 0.6 | **1,0** | **1,0** | **0.7** | 0.4 | 0 | 0 | 0.1 | 0 |
| 2-029D05 | NM_006925 | splicing factor, arginine/serine-rich 5 | SFRS5 | [6430](http://www.ncbi.nlm.nih.gov/entrez/query.fcgi?db=gene&cmd=Retrieve&dopt=Graphics&list_uids=6430) | 4 | **1.2** | **1.5** | **2,0** | **1.1** | **0.8** | -0.1 | **0.9** | 0.5 | 0.6 |
| 2-033G01 | NM_031263 | heterogeneous nuclear ribonucleoprotein K | HNRPK | 3190 | 3 | **0.8** | **0.8** | **0.8** | 0.5 | -0.1 | -0.2 | -0.1 | 0.1 | 0.1 |
|  |  | ***Others*** |  |  |  |  |  |  |  |  |  |  |  |  |
| 2-033A11 |  | Unknown |  |  | 5 | **0.8** | **0.8** | **0.9** | **0.7** | 0.4 | -0.1 | 0.3 | 0.1 | 0.1 |
| BL-009E04 |  | Unknown |  |  | 4 | **0.8** | **1.6** | **1.1** | **0.9** | 0.4 | -0.2 | 0.4 | 0.3 | 0.1 |
| 2-029E07 |  | Unknown |  |  | 3 | **1.1** | 0.3 | **1.9** | **1.6** | 0.2 | 0 | 0.1 | -0.1 | 0.3 |
| 2-030D10 |  | Unknown |  |  | 3 | **0.8** | **1.6** | **1.6** | **2.2** | **0.9** | **-1.2** | -0.6 | 0.1 | 0.2 |
| 2-024C05 | NM_006169 | nicotinamide N-methyltransferase | NNMT | 4837 | 3 | **1,0** | **1.4** | **1.5** | **1.2** | **1.1** | 0 | 0.2 | 0.5 | 0.2 |
| **Under expressed genes** | | | | | | **Log_2_ ratio intensities** | | | | | | | | |
|  |  | ***Cellular metabolism*** |  |  |  |  |  |  |  |  |  |  |  |  |
| **2-002F10** | **NM_016311** | ATPase inhibitory factor 1 | ATPIF1 | 93974 | -7 | -0.5 | **-0.9** | **-0.7** | **-0.7** | 0.1 | 0 | 0.2 | 0 | 0.4 |
| 2-026D10 | NM_016029 | dehydrogenase/reductase (SDR family) member 7 | DHRS7 | 51635 | -7 | **-1.2** | **-1.4** | **-1.1** | -0.5 | 0.2 | 0.3 | 0.3 | 0.2 | 0.1 |
| 2-035A10 | NM_005061 | ribosomal protein L3-like | RPL3L | 6123 | -5 | **-0.8** | **-1,0** | **-0.9** | -0.6 | -0.1 | 0.1 | -0.3 | -0.3 | 0.1 |
| 2-026B03 | NM_020707 | glycerol uptake/transporter homolog | GUP1 | 57467 | -5 | **-0.8** | **-1.1** | **-0.7** | **-0.7** | 0 | 0.1 | -0.1 | -0.4 | -0.1 |
| 2-036B10 | NM_033500 | hexokinase 1 isoform HKI-td, | HK1 | 3098 | -5 | -0.5 | **-0.8** | **-0.8** | **-0.7** | -0.1 | 0.3 | 0 | 0.1 | -0.2 |
| 2-010H09 | NM_003000 | succinate dehydrogenase complex, subunit B, iron sulfur | SDHB | 6390 | -3 | **-1.1** | **-1.2** | **-1,0** | -0.6 | -0.6 | -0.2 | -0.5 | -0.4 | -0.1 |
| 2-017E02 | NM_152246 | carnitine palmitoyltransferase 1B | CPT1B | 1375 | -3 | **-0.7** | **-0.8** | **-0.8** | -0.5 | 0.2 | -0.4 | -0.2 | -0.4 | 0 |
| 2-026D03 | NM_000365 | triosephosphate isomerase 1 | TPI1 | 7167 | -3 | **-0.9** | **-0.9** | **-0.8** | -0.5 | -0.4 | 0.1 | -0.3 | -0.5 | -0.4 |
| 2-020E03 | NM_174869  NM_004135 | isocitrate dehydrogenase 3 (NAD+) gamma | IDH3G | 3421 | -3 | -0.3 | **-0.7** | **-0.8** | **-0.8** | -0.2 | -0.1 | 0 | -0.5 | -0.2 |
| 2-029C05 | NM_015141 | glycerol-3-phosphate dehydrogenase 1-like | GPD1L | 23171 | -3 | **-1,0** | **-1.2** | **-0.9** | -0.5 | -0.1 | 0.1 | **-0.8** | -0.3 | 0.4 |
| 2-010H11 | NM_000019 | acetyl-Coenzyme A acetyltransferase 1 | ACAT1 | 38 | -3 | **-1.1** | **-0.9** | **-0.7** | -0.6 | -0.3 | 0.1 | **-0.7** | -0.3 | -0.1 |

**Table 5 (continuation):** Genes differentially expressed between SMA I and SMA III muscles.

| **Microarray**  **ID** | **Ref. Seq.** | **Gene name and**  **functional category** | **Gene**  **Symbol** | **Entrez**  **Gene ID** | **SAM**  **Score** | **SMA I biopsy code** | | | | **SMA III biopsy code** | | | | |
| --- | --- | --- | --- | --- | --- | --- | --- | --- | --- | --- | --- | --- | --- | --- |
|  |  |  |  |  |  | **A** | **B** | **C** | **D** | **E** | **F** | **G** | **H** | **I** |
| **Under expressed genes** | | | | | | **Log_2_ ratio intensities** | | | | | | | | |
|  |  | *Muscle contraction* |  |  |  |  |  |  |  |  |  |  |  |  |
| 2-020G12 | NM_001824 | muscle creatine kinase | CKM | 1158 | -6 | **-0.7** | **-1.3** | **-1.1** | **-0.9** | 0 | -0.2 | -0.4 | -0.3 | 0.1 |
| 2-018E09 | NM_005963 | myosin, heavy polypeptide 1, skeletal muscle | MYH1 | 4619 | -4 | **-0.8** | **-1.6** | **-0.9** | **-1.2** | 0.1 | -0.3 | 0.6 | -0.4 | 0.4 |
| 2-020F04 | NM_001824 | muscle creatine kinase | CKM | 1158 | -3 | **-0.9** | **-1.6** | **-1.3** | **-1.3** | -0.5 | -0.1 | -0.3 | **-1.1** | 0 |
| 2-001A02 | NM_001824 | muscle creatine kinase | CKM | 1158 | -3 | **-0.8** | **-1.5** | **-0.9** | **-1,0** | -0.5 | -0.1 | -0.2 | -0.7 | 0 |
| 2-021D12 | NM_001824 | muscle creatine kinase | CKM | 1158 | -3 | -0.5 | **-1.5** | **-1.3** | **-1.1** | -0.6 | -0.1 | -0.2 | **-0.7** | -0.2 |
| 2-023A02 | NM_001824 | muscle creatine kinase | CKM | 1158 | -3 | -0.5 | **-1.7** | **-1.3** | **-1,0** | -0.6 | -0.3 | -0.2 | -0.6 | -0.3 |
| 2-010G04 | NM_001825 | sarcomeric mitochondrial creatine kinase | CKMT2 | 1160 | -3 | **-1.1** | **-1.9** | **-0.9** | -0.6 | -0.2 | 0 | 0 | -0.5 | -0.3 |
| 2-020B02 | NM_001927 | desmin | DES | 1674 | -3 | **-0.7** | **-1.3** | **-0.7** | **-0.7** | 0 | 0.3 | 0 | -0.6 | 0.1 |
|  |  | ***Signal transduction*** |  |  |  |  |  |  |  |  |  |  |  |  |
| 2-030B08 | NM_004221 | interleukin 32 | IL32 | 9235 | -5 | **-0.9** | **-0.8** | **-0.8** | **-1,0** | 0 | 0.1 | 0.1 | -0.4 | 0 |
| 2-037B08 | NM_145687  NM_145686  NM_004834 | mitogen-activated protein kinase kinase kinase kinase 4 | MAP4K4 | 9448 | -4 | **-0.9** | **-1.2** | **-1.1** | **-0.8** | -0.4 | 0 | -0.1 | -0.5 | 0 |
| 2-023C11 | NM_003732 | eukaryotic translation initiation factor 4E binding protein 3 | EIF4EBP3 | 8637 | -4 | **-0.9** | **-1,0** | **-0.8** | -0.3 | 0.3 | 0.1 | 0.1 | 0.2 | 0.1 |
| 2-040C12 | NM_199054 | MAP kinase interacting serine/threonine kinase 2 | MKNK2 | 2872 | -3 | **-1.5** | **-2,0** | **-1.2** | -0.2 | -0.2 | 0.1 | -0.2 | -0.5 | 0.2 |
|  |  | ***Molecular recognition system*** |  |  |  |  |  |  |  |  |  |  |  |  |
| 2-033A04 | NM_022778 | coiled-coil domain containing 21 | CCDC21 | 64793 | -4 | **-1.1** | **-1.1** | **-1.1** | -0.6 | 0 | 0 | -0.4 | **-0.7** | -0.1 |
| 2-005A06 | NM_206860 | transforming, acidic coiled-coil containing protein 2 | TACC2 | 10579 | -4 | **-0.9** | **-1.1** | **-0.7** | -0.4 | 0 | -0.1 | -0.1 | -0.4 | 0.3 |
| 2-028C07 | NM_080647 | T-box 1 isoform C,T-box 1 isoform AT-box 1 isoform B | TBX1 | 6899 | -3 | **-0.8** | **-1.2** | **-1.1** | **-0.8** | 0 | 0.1 | 0.1 | -0.2 | -0.2 |
|  |  | ***Cell cycle*** |  |  |  |  |  |  |  |  |  |  |  |  |
| 2-037H12 | NM_173478 | cyclin N-terminal domain containing | CNTD | 124817-8678 | -3 | **-1.2** | **-1.2** | **-0.9** | -0.5 | -0.4 | 0 | -0.3 | -0.5 | 0.2 |
|  |  | ***Others*** |  |  |  |  |  |  |  |  |  |  |  |  |
| 2-029F08 | NM_014367 | chromsome 3 open reading frame 28 | C3orf28 | 26355 | -6 | **-1.2** | **-1,0** | **-0.8** | -0.6 | -0.3 | -0.1 | -0.3 | -0.1 | 0.1 |
| 2-010B01 | NM_032747 | upregulated during skeletal muscle growth 5 | USMG5 | 84833 | -4 | **-0.8** | **-1,0** | **-0.7** | -0.4 | -0.2 | -0.2 | -0.1 | 0.1 | -0.1 |
| 2-021E01 | NM_002856 | poliovirus receptor-related 2 (herpesvirus entry mediator B) | PVRL2 | 5819 | -3 | -0.5 | **-1.3** | **-1.1** | **-1.2** | -0.6 | 0 | -0.3 | -0.6 | 0.1 |
| 2-026H06 |  | Unknown |  |  | -3 | -0.5 | **-1.3** | -0.3 | -0.1 | **1.5** | **1.6** | **0.7** | -0.1 | -0.2 |
