# Supplementary material for: Different atrophy-hypertrophy transcription pathways in muscles affected by severe and mild spinal muscular atrophy
Source: BMC Med. 2009 Apr 7;7:14. doi: 10.1186/1741-7015-7-14 (PMC2676312; doi:10.1186/1741-7015-7-14)
Supplement: Additional File 7 — Additional Table S6. This table contains the list of genes with an altered expression values in both SMA I and SMA III muscles, in comparison to normal controls. [file 1741-7015-7-14-S7.doc]

**Table 6.** Genes with an altered expression values in both SMA I and SMA III muscles, in comparison to normal controls.

| **Microarray**  **ID** | **Ref. Seq.** | **Gene name and**  **functional category** | **Gene**  **Symbol** | **Entrez**  **Gene ID** | **SAM**  **Score** | **SMA I biopsy code** | | | | **SMA III biopsy code** | | | | |
| --- | --- | --- | --- | --- | --- | --- | --- | --- | --- | --- | --- | --- | --- | --- |
|  |  |  |  |  |  | **A** | **B** | **C** | **D** | **E** | **F** | **G** | **H** | **I** |
| **0ver expressed genes** | | | | | | **Log_2_ ratio intensities** | | | | | | | | |
|  |  | ***Signal transduction*** |  |  |  |  |  |  |  |  |  |  |  |  |
| 2-010C12 | NM_014624 | S100 calcium-binding protein A6 | S100A6 | 6277 | 6 | **1.1** | **2.1** | **1.8** | 0.6 | **1.1** | 0.5 | **1** | **1.6** | **1.1** |
| 2-038C09 | NM_138822 | peptidylglycine alpha-amidating monooxygenase | PAM | 5066 | 6 | **1.4** | **1.7** | **1.7** | **0.9** | **1.1** | 0.2 | 0.6 | **1** | **0.9** |
|  |  | *Transport* |  |  |  |  |  |  |  |  |  |  |  |  |
| 2-030H05 | NM_006407 | ADP-ribosylation-like factor 6 interacting protein 5 | ARL6IP5 | 10550 | 7 | **1.1** | **1.2** | **1.4** | **0.9** | **0.9** | 0.1 | **0.8** | **1.1** | **0.9** |
| 2-030F11 | NM_003746 | dynein, light chain, LC8-type 1 | DYNLL1 | 8655 | 5 | **1.7** | **2.2** | **1.1** | **0.9** | **0.9** | -0.2 | **0.7** | **1.1** | **0.9** |
|  |  | ***RNA metabolism*** |  |  |  |  |  |  |  |  |  |  |  |  |
| 2-020C09 | NM_001402 | eukaryotic translation elongation factor 1 alpha 1 | EEF1A1 | 1915 | 6 | **2** | **2** | **2.1** | **1.8** | **1.9** | -0.1 | **1.6** | **1.4** | **1.4** |
| 2-029C10 | NM_031844 | heterogeneous nuclear ribonucleoprotein U isoform a | HNRPU | 3192 | 5 | **1.2** | **1.4** | **1.5** | **1.4** | **0.9** | -0.1 | **1** | **0.7** | **0.9** |
|  |  | ***Molecular recognition system*** |  |  |  |  |  |  |  |  |  |  |  |  |
| 2-036H10 | NM_198494 | zinc finger protein 642 | ZNF642 | 339559 | 6 | **1** | **1.8** | **1** | **0.9** | **0.7** | 0.2 | **0.8** | **1** | 0.6 |
|  |  | ***Immune/Defense response*** |  |  |  |  |  |  |  |  |  |  |  |  |
| 2-011B07 | NM_002113 | complement factor H | CFH | 3075 | 5 | **2.2** | **3.2** | **2.9** | **2.3** | **0.9** | 0.5 | **1** | **0.8** | **1** |
| 2-029D06 | NM_201442 | complement component 1, s subcomponent | C1S | 716 | 4 | **0.9** | **3** | **1.5** | **1.7** | **1.1** | 0.3 | 0.6 | **1.1** | **1** |
|  |  | ***Others*** |  |  |  |  |  |  |  |  |  |  |  |  |
| 2-036A04 |  | Unknown |  |  | 5 | **1** | **1.6** | **1.5** | 0.2 | **1.3** | 0.3 | **1.1** | **1.5** | **0.8** |
| 2-031F07 | NM_018464 | chromosome 10 open reading frame 70 | C10orf70 | 55847 | 5 | **0.9** | **1.5** | **1** | **0.9** | 0.5 | -0.1 | **0.8** | **1.1** | **1.1** |
| 2-002C07 | NM_002954 | ribosomal protein S27a | RPS27A | 6233 | 5 | **1.1** | **1.3** | **1.6** | **0.9** | **0.9** | 0 | **0.7** | **0.7** | 0.3 |
| **Under expressed genes** | | | | | | **Log_2_ ratio intensities** | | | | | | | | |
|  |  | ***Cellular metabolism*** |  |  |  |  |  |  |  |  |  |  |  |  |
| 2-002B09 | NM_002168 | isocitrate dehydrogenase 2 (NADP+), | IDH2 | 3418 | -5 | **-1.7** | **-2.1** | **-1.7** | **-1.3** | **-0.9** | -0.1 | **-0.8** | **-1.1** | -0.5 |
| 2-020B09 | NM_152328 | adenylosuccinate synthase-like 1 | ADSSL1 | 122622 | -5 | **-1.9** | **-2.5** | **-2.5** | **-1.8** | **-1.1** | -0.2 | **-0.8** | **-1.3** | -0.4 |
| 2-015C07 | NM_001151 | solute carrier family 25 (mitochondrial carrier), member 4 ANT1 | SLC25A4 | 291 | -4 | **-2.5** | **-3.3** | **-2.7** | **-2.1** | **-0.9** | -0.4 | **-1.5** | **-1.1** | -0.2 |
|  |  | *Muscle contraction* |  |  |  |  |  |  |  |  |  |  |  |  |
| 2-002A07 | NM_004468 | four and a half LIM domains 3 | FHL3 | 2275 | -4 | **-1.6** | **-2.2** | **-1.9** | **-0.8** | **-0.7** | 0.1 | **-0.7** | **-1** | **-0.8** |
| 2-010B10 | NM_133437 | titin | TTN | 7273 | -4 | **-0.9** | **-1.9** | **-1.5** | **-1.7** | **-1.3** | 0.2 | -0.3 | **-1** | **-0.8** |
|  |  | ***Transport*** |  |  |  |  |  |  |  |  |  |  |  |  |
| BL-008B07 | NM_003234 | transferrin receptor (p90, CD71) | TFRC | 7037 | -5 | **-1.2** | **-1.4** | **-2** | **-1.4** | -0.6 | 0.2 | **-1.1** | **-0.9** | **-1.7** |
|  |  | ***Others*** |  |  |  |  |  |  |  |  |  |  |  |  |
| 2-001F01 | NM_213720 | chromosome 22 open reading frame 16 | C22orf16 | 400916 | -5 | **-1.6** | **-1.8** | **-1.4** | **-1.1** | -0.5 | 0 | **-0.8** | **-0.7** | **-0.7** |
